# Inheritance flows in Switzerland, 1911–2011

**DOI:** 10.1186/s41937-017-0012-9

**Published:** 2018-03-06

**Authors:** Marius Brülhart, Didier Dupertuis, Elodie Moreau

**Affiliations:** 1 University of Lausanne, Lausanne, Switzerland; 2 VZ VermögensZentrum, Geneva, Switzerland; 3 Department of Economics, Faculty of Business and Economics (HEC), University of Lausanne, Lausanne, 1015 Switzerland; 4 Centre for Economic Policy Research, London, UK

**Keywords:** Inheritance, Switzerland, D31, H24, N34

## Abstract

We estimate the size of inheritance flows in Switzerland over a long span of data, in close analogy to the study for France by Piketty (Q J Econ 126(3):1071–1131, 2011). We find that inheritance flows had been growing more slowly than national income up until the 1970s, but have been outpacing income growth since. According to our central estimates, the annual flow of inheritance amounted to 13.2% of national income in 2011. The share of total wealth that is attributable to inheritance has remained relatively stable over time, fluctuating between 45 and 60%.

## Background

Interest in inheritance has recently been revived both in policy debates and in the scientific community. Policy makers’ prime concern is with the taxation of bequests—one of the most emotionally and ideologically charged areas of public finance. This debate is of increasing interest also to economic researchers. Wealth inequality, after a prolonged contraction over the twentieth century, has been rising again since the 1980s, and inheritance may be an important channel shaping that trend (Piketty and Zucman 2014). Moreover, apart from distributional concerns, the larger are aggregate bequest flows, the more strongly they should feature in tax policies that aim at broad tax bases.

Despite this evident interest in quantifying the economic importance of inheritance, direct measures have been constructed only rather recently. Piketty (2011) reports a 200-year time series for France, showing that the weight of inheritance—meaning the sum of bequests at death and gifts *inter vivos*—is growing strongly and approaching levels not seen since the early twentieth century. Similar long-run evolutions have been documented for Germany (Schinke 2012), the UK (Atkinson 2013) and Sweden (Ohlsson et al. 2016).

We construct corresponding data series for Switzerland, which represents an interesting comparison country, especially as it was spared the mass destructions of the two world wars. If we did not observe the u-shaped evolution of inheritance over the last century that have been found for France and Germany, or if the “u” were less pronounced in Switzerland, the attribution to war destruction of those countries’ twentieth-century dips in the weight of inheritance would be corroborated^1^. Moreover, Swiss policy discussions about bequest taxation often imply assertions about the perceived level and trend in the importance of inheritance, but to date, no time-series evidence exists on the subject.

We find that the size of inheritance flows relative to total wealth and to total income in Switzerland is relatively high compared to France and particularly Germany. However, Switzerland also appears to have witnessed a dip in inheritance in the first half of the twentieth century, suggesting that war destruction may not be the only, or even the main, reason behind the u-shaped evolutions in other European nations. Moreover, since the 1980s, Switzerland seems to be witnessing an increase in the importance of inheritance that is comparable to other mature economies.

The paper is organized as follows. The “Measurement and data” section describes the estimation method and outlines our data sources. In the “Results” section, we show the results, and the “Conclusions” section concludes.

## Measurement and data

### Basic definitions

To the extent that our data allow us to, we follow Piketty (2011) by estimating “economic inheritance flows”^2^.

Specifically, our first measure of interest is the share of total private wealth that is transferred through inheritance in any given year, where we understand “inheritance” to comprise wealth transfers both at death and *inter vivos*. That share is defined by the following simple accounting equation:

1 $$ b_{wt}=\frac{B_{t}}{W_{t}}=m_{t}\cdot \mu_{t}^{\ast},  $$

where *b*_*wt*_ is the inheritance-to-wealth ratio, *B*_*t*_ stands for the the sum of private capital transfers between generations (“*B*equests”) in a particular year *t*, *W*_*t*_ is aggregate private wealth, *m*_*t*_ stands for the mortality rate over the adult population (defined as 20 years or older), and $\mu _{t}^{\ast }$ is the gift-adjusted ratio between average adult wealth at death and the average wealth of the living^3^.

Unless we factor in gifts *inter vivos*, wealth transfers at death will understate life-time wealth transfers. The measure of the relative wealth at death is therefore adjusted for gifts in the following way:

$$\mu_{t}^{\ast}=(1+v_{t})\cdot \mu_{t}, $$

where *v*_*t*_ represents the ratio of gifts *inter vivos*, *V*_*t*_, to total bequest flows $\left (v_{t}=\frac {V_{t}}{B_{t}}\right)$, and *μ*_*t*_ is the unadjusted ratio between average wealth at death and the average wealth of the living.

As a complement to *b*_*wt*_, which compares the flow of inheritance to the stock of wealth, we also report the ratio *b*_*yt*_, which scales the flow of inheritance to the flow of aggregate income:

2 $$ b_{yt}=\frac{B_{t}}{Y_{t}}=m_{t}\cdot \mu_{t}^{\ast}\cdot \frac{W_{t}}{Y_{t}},  $$

where *Y*_*t*_ is net national income.

Armed with an estimate of *b*_*yt*_, we can finally compute the share of inherited wealth in the stock of wealth according to the following equation, due to Piketty and Zucman (2015) and Alvaredo et al. (2017):

3 $$ \phi_{t}=\frac{b_{yt}}{b_{yt}+(1-\alpha_{t})\cdot s_{t}},  $$

where *α* denotes capital’s share of national income (the remainder 1−*α*_*t*_ accruing to labour), and *s*_*t*_ stands for the saving rate. Intuitively, all wealth has to originate either in inheritance or in savings out of labour income. Hence, this measure expresses the flow of inheritance relative to the flow of inheritance plus total savings out of labour income. Implicit is the assumption that the propensity to save out of labour income is equal to the propensity to save out of capital income. As the latter is probably higher due to the more unequal distribution of wealth compared to labour income, *ϕ*_*t*_ yields conservative estimates of the weight of bequests.

Wealth is built over the life cycle. Hence, *b*_*yt*_,*s*_*t*_ and *α*_*t*_ are averaged over 30 years, the typical length of a generation, to account for past variations in savings and income that affect present inherited and accumulated wealth.

Note that *ϕ*_*t*_ as defined by Eq. (3) is quite different from (and in important respects more informative than) *b*_*wt*_ defined in Eq. (1). *b*_*wt*_ reports the *flow* of bequests as a share of the stock of wealth in a given year, whereas *ϕ*_*t*_ compares the capitalized *stock* of bequests to the stock of wealth. The measure *ϕ*_*t*_ therefore tells us how much of an average franc of wealth is inherited as opposed to being “self made”.

### Estimating $\protect \mu _{t}^{\ast }$

The empirically most demanding element of Eqs. (1) and (2) is $\mu _{t}^{\ast }$, as Swiss data do not allow us to observe wealth at death. We therefore take an indirect approach, by first estimating age-wealth profiles of the living, and then deriving age-wealth profiles at death^4^.

Based on tax statistics for Zurich, Switzerland’s most populous canton, we know the number of taxpayers per year *t*, wealth bracket *ω* and age group *a*, *T*_*t,a*,*ω*_, as well as total wealth per year and wealth bracket, *W*_*t*,*ω*_. We thus compute average wealth per wealth bracket as $w_{t,\omega }=\frac {W_{t,\omega }}{\sum _{a}(T_{t,a,\omega })}$. Assuming within-bracket averages to be constant across age groups, this allows us to recover age-wealth profiles as follows:

4 $$w_{t}(a)=\frac{\sum_{\omega}(w_{t,\omega}\cdot T_{t,a,\omega})}{\sum_{\omega}(T_{t,a,\omega})}.  $$

Second, we estimate age-wealth profiles at death, by combining wealth-dependent mortality rates with our estimated age-wealth profiles of the living. We distinguish between the poor, *p*, with wealth below the median, and the rich, *r*, with above-median wealth. The poor tend to have higher mortality rates than the rich at all age groups *a*$\left (m_{t}^{p}(a)\geq m_{t}^{r}(a)\right)$, but the mortality differential typically decreases with age^5^.

Average mortality per age group is given by

$$m_{t}(a)=\frac{N_{dt}(a)}{N_{t}(a)}=\frac{m_{t}^{p}(a)+m_{t}^{r}(a)}{2}, $$

where *N*_*dt*_(*a*) is the number of deaths in year *t* and age group *a*, and *N*_*t*_(*a*) is the corresponding number of living individuals, and the simple average being due to the fact that the rich-poor split is placed at the median.

Define $sh_{t}^{p}(a)$ as the share of total wealth owned by the poor in year *t*, per age group *a*. Unlike Piketty (2011), we are able to calculate $sh_{t}^{p}(a)$ for every age group. We estimate this share using linear interpolation on age-dependent wealth distributions for the canton of Zurich^6^. Combined with (4), this allows us to estimate, for each age group *a*, the average wealth of the poor $w_{t}^{p}(a)$ and of the rich $ w_{t}^{r}(a)$, respectively^7^:

$$w_{t}^{p}(a)=2\cdot sh_{t}^{p}(a)\cdot w_{t}(a),\text{and}\ w_{t}^{r}(a)=2\cdot \left(1-sh_{t}^{p}(a)\right)\cdot w_{t}(a). $$

Hence, we can compute average wealth at death of age group *a* as follows:

5 $$ \begin{aligned} w_{dt}(a)& \,=\,\frac{w_{t}^{p}(a)\cdot m_{t}^{p}(a)+w_{t}^{r}(a)\cdot m_{t}^{r}(a)}{m_{t}^{p}+m_{t}^{r}}  \\ &\! =\!\frac{\left[2\!\cdot \! sh_{t}^{p}(a)\!\cdot \! w_{t}(a)\cdot m_{t}^{p}(a)\right]\,+\,\left[2\cdot \left(1-sh_{t}^{p}(a)\!\right)\cdot w_{t}(a)\cdot m_{t}^{r}(a)\right]}{m_{t}^{p}+m_{t}^{r}}. \end{aligned}  $$

With age-wealth profiles both at death and overall thus defined, we can recover the aggregate ratio of wealth at death over wealth of the living, *μ*_*t*_, as follows:

$$\mu_{t}= \frac{\sum_{a} w_{dt}(a)\cdot N_{dt}(a)}{\sum_{a} w_{t}(a)\cdot N_{t}(a)}. $$

### Data

We employ a number of complementary data sources, some covering the entire country and some being based on subsets of cantons. Details are provided in the Appendix.

Our data for private wealth are drawn from country-wide official wealth statistics based on tax declarations, dating back to 1913. We adjust those data for the 30% undervaluation of real estate that seems to be an enduring feature of Swiss taxation and for tax-exempt pension assets that are withdrawn as lump-sum payouts upon retirement^8^. Net national income (*Y*_*t*_) is available from a variety of sources back to 1906.

**Fig. 1:**
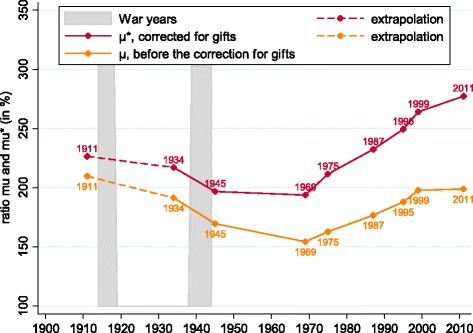
Evolution of the ratio of wealth at death and wealth while alive (uncorrected for gifts, *μ*_*t*_ and corrected for gifts, $\mu _{t}^{*}$). Notes: see text for data sources, and Table 1 for data points

Age-wealth profiles for the computation of *μ*_*t*_, the ratio of wealth at death over average wealth while alive, are based on tax records for the canton of Zurich that reach back to 1934. Looking at Fig. 1 (data points in Table 1), we observe a u-shaped evolution of *μ*_*t*_ over the course of the last century, similar to that observed in other nations. Zurich-specific age-wealth profiles should offer representative measures for the country as a whole, given that prior research has shown that correcting for differing age distributions across cantons makes a negligible difference to the estimates (see Daepp 2003, p. 21).

**Table 1:** Data points for estimated Swiss inheritance series

	*μ*_*t*_ in % (Fig. 1)	$\mu _{t}^{*}$ in % (Fig. 1)	*b*_*wt*_ in % (Fig. 3)	*b*_*yt*_ in % (Fig. 6)
1911	209.7	226.4	4.23	16.2
1934	191.3	217.1	3.20	11.1
1945	169.5	196.8	2.87	8.9
1969	154.2	193.7	2.50	6.4
1975	162.7	211.5	2.55	6.5
1987	176.7	232.4	2.66	7.4
1995	187.8	249.5	2.88	8.7
1999	197.9	264.2	2.96	10.8
2011	198.8	277.4	2.77	13.2

**Fig. 2:**
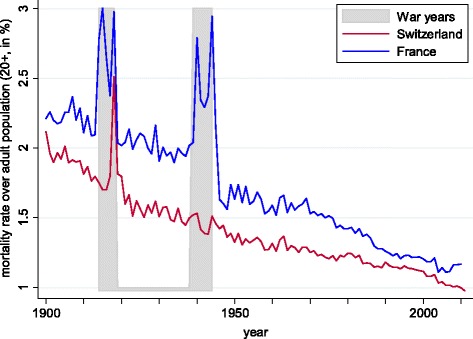
Adult mortality rates. Notes: data for France from Piketty (2011). See text for Swiss data sources

Adult mortality rates are taken from a variety of sources, stretching back to 1900. Our mortality series for Switzerland is shown in Fig. 2, together with the corresponding data for France taken from Piketty (2011). Mortality decreased steadily from 2.1% in 1900 to 1% in 2011^9^. Moreover, the Swiss mortality rate has consistently been lower than that of France, although the two series appear to be converging.

The weight of *inter vivos* gifts for the computation of the gift-corrected average wealth at death relative to wealth of the living, $\mu _{t}^{\ast }$, is computed from a sample of cantons for which this information is available back to 1995, and extrapolated to 1911 based on the evolution of recorded *inter vivos* gifts in Germany. We show our constructed $\mu _{t}^{\ast }$ in Fig. 1. It is apparent that the importance of *inter vivos* gifts has been growing over time.

We also note that, at 277% in 2011, the $\mu _{t}^{\ast }$ we compute is large. The average Swiss at the time of death possesses close to three times as much wealth as the average living Swiss person. Piketty (2011) estimates this value at 223% for France in 2008, and Ohlsson et al. (2016) compute a value of 162% for Sweden in 2010. This is a key driver of the comparatively large inheritance sizes we report below.

## Results

### Inheritance flows relative to private wealth

**Fig. 3:**
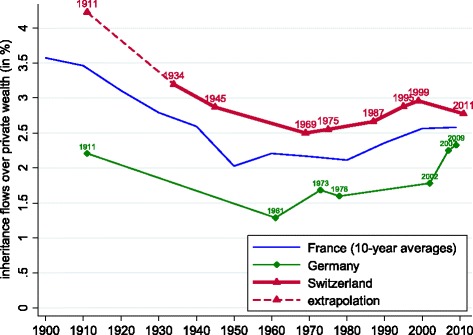
Annual inheritance flow as a fraction of private wealth: Switzerland, France and Germany. Notes: Data for France from Piketty (2011). Data for Germany from Schinke (2012). See text for Swiss data sources, and Table 1 for data points

In Fig. 3, we track how the annual inheritance flow as a share of the stock of private wealth (*b*_*wt*_) has evolved over the last century. For comparison, we also show the corresponding series for France and Germany. Our estimates show that Switzerland has broadly shared in the u-shaped evolution of inheritance observed in other European nations over the twentieth century. While, scaled to total private wealth, inheritance flows have historically been bigger in Switzerland, our calculations suggest a process of convergence over the last decade. In the logic of Eq. (1), the recent decrease in *b*_*wt*_ results from mortality falling more strongly (Fig. 2) than the rise in $\mu _{t}^{\ast }$, the ratio between wealth at death (including previously made gifts) and wealth of the living (Fig. 1).

### Inheritance flows relative to national income

**Fig. 4:**
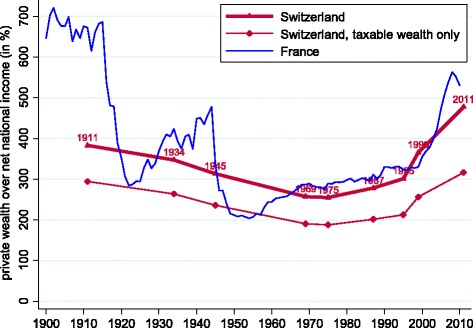
Private wealth as a fraction of net national income: Switzerland and France. Notes: data for France from Piketty (2011). See text for Swiss data sources

It is of interest to scale inheritance flows not only to the stock of wealth but also to the flow of income. This is given by the ratio *b*_*yt*_ of Eq. (2). The computation of this ratio requires us to enlist also data on aggregate wealth and net incomes. We therefore begin by presenting what our data construction described in the “Data” section implies for wealth-to-income ratios in Switzerland $\left (\frac {W_{t}}{Y_{t}}\right)$. These estimates are reported in Fig. 4. The graph clearly shows how our tax-based wealth data series is consistently lower than our preferred series that is adjusted for undervalued real estate and omitted pension fund assets. We observe that wealth-to-income ratios have shown a steep increase since the 1970s and have been approaching 500% in the most recent sample year. In that respect, Switzerland conforms to a trend shared by all the mature economies for which we have comparable data (Piketty and Zucman 2014)^10^.

**Fig. 5:**
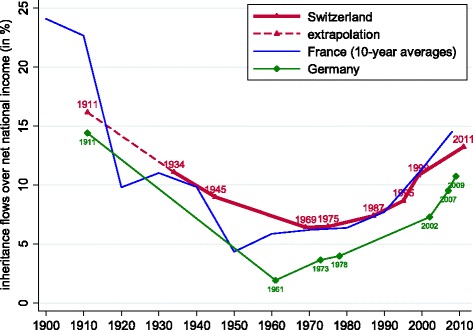
Annual inheritance flow as a fraction of national income: Switzerland, France and Germany. Notes: data for France averaged by decade from Piketty (2011). Data for Germany from Schinke (2012). See text for Swiss data sources, and Table 1 for data points

In Fig. 5, we show the evolution of the inheritance-to-income ratio estimated for Switzerland, together with comparable series for France and Germany. When expressed in this way (rather than when scaled to wealth), we observe a continuing marked increase in inheritance over the four decades up to the end of our sample in 2011. The ratio *b*_*yt*_ fell from 16.2% in 1911 to 6.4% in 1969 and then rose again to 13.2% in 2011. Relative to the flow of income, therefore, intergenerational transfers now appear to be more than twice as important as they were half a century ago, and close to the level last seen in the early 1900s.

**Fig. 6:**
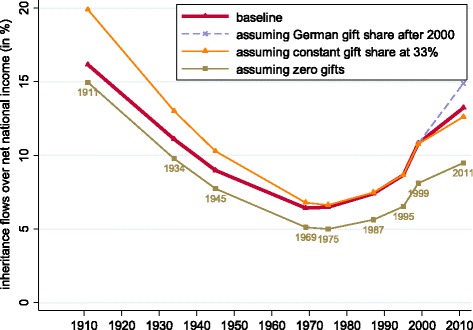
Annual inheritance flow as a fraction of national income: robustness to assumptions on *inter vivos* gifts. Notes: see text for data sources

As a robustness check, we compare our baseline estimated series of *b*_*yt*_, reported in Fig. 5, to corresponding data series based on varying assumptions on *inter vivos* gifts. Figure 6 shows that our qualitative findings are not affected by our baseline assumptions in this respect.

### The stock of inherited wealth as a share of total wealth

**Fig. 7:**
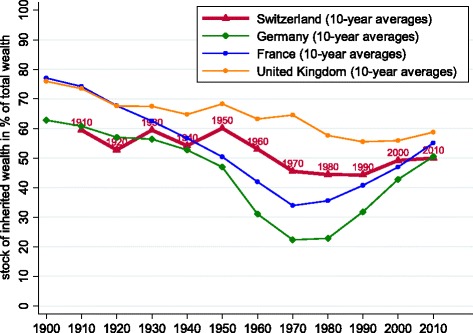
Cumulative stock of inheritances as a fraction of private wealth. Notes: data points are 30-year moving averages, reported every 10 years. See text for Swiss data sources. Data for France and Germany from Alvaredo et al. (2017), data for the United Kingdom are from Atkinson (2013)

In Fig. 7, we show the evolution of *ϕ*, the share of total wealth that can be attributed to past inheritance, computed using Eq. (3) with 30-year moving averages. According to our calculations, this share has historically fluctuated in an interval roughly between 45 and 60%, with less of a pronounced u-shape than those observed in France and Germany. We observe an increasing trend in *ϕ* since 1990, with an estimated value of 0.50 in 2010. Given that this method likely underestimates the weight of inheritance (as it imputes too high a share of savings to labour earnings; see Piketty and Zucman 2015), our computations suggest that at least half of Swiss private wealth has been acquired through inheritance.

**Fig. 8:**
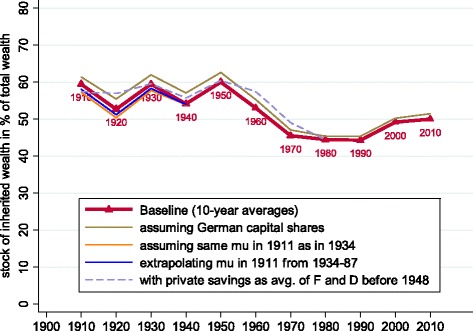
Cumulative stock of inheritances as a fraction of private wealth: robustness to alternative assumptions. Notes: data points are 30-year moving averages, reported every 10 years. See text for data sources

As discussed in the “Capital shares and saving rates” section in the Appendix, the historical data on capital shares and saving rates that underly Fig. 7 might not be perfectly precise. We have therefore explored the implications of using alternative data approximations. The main variants are illustrated in Fig. 8. We find that our estimated *ϕ* are not much changed by alternative approximations.

## Conclusions

We have reported estimates of inheritance flows in Switzerland from 1911 to 2011. The annual volume of inheritance flows relative to total wealth and to total income is relatively high in Switzerland, compared to France and particularly Germany. Switzerland has witnessed a similar u-shaped evolution of the weight of inheritance flows over the past century, and it seems to be experiencing an increase in the inheritance-to-income ratio that is comparable to other mature economies in the early twenty-first century. This increase appears to be driven mainly by an increase in the wealth-to-income ratio but also partly by a growing weight of inheritance as a wealth acquisition channel^11^.

Due to an absence of federal-level inheritance tax data, our analysis had to be based on estimating “economic inheritance flows”, requiring some strong assumptions particularly for the early part of our sample period. Given that bequests have long been taxed in a majority of cantons, it might therefore be worthwhile investigating further if some cantonal archives offer more detailed long-term data on gifts and inheritances. Cantonal bequest-tax data might allow researchers to track the evolution not only of the volume of inheritances but also of the distribution across bequest sizes and heir categories. Such information is essential for optimal policy design but remains beyond the reach of the data material currently at our disposal^12^.

Finally, an important ingredient to our computed inheritance series are estimates of $\mu _{t}^{\ast }$, the ratio of average wealth at death over wealth of the living. This ratio seems to be large in Switzerland compared to other countries and would therefore merit further investigation. We have to leave this issue to future research as well.

## Appendix

## Data sources

### National income and private wealth

In order to estimate inheritance-to-income ratios *b*_*yt*_, we need to find data on two additional variables: net national income (*Y*_*t*_) and aggregate bequeathable private wealth (*W*_*t*_); see Eq. (2).

For *Y*_*t*_, we use data series for net national income (NNI), which is gross national income (GNI) minus the consumption of fixed capital. In turn, GNI equals GDP minus primary incomes payable to non-residents, plus primary incomes receivable from non-residents. For the period 1906–1938, we use the NNI estimates reported by Andrist et al. (2000). For the period 1938–1956, the relevant information can be found in the *Annuaire Statistique Suisse* 1957 (p. 347). For the period 1965–1995, the data are obtained from the Federal Statistical Office. Since that series stops in 1995, we use data from OCSTAT Geneva (years 1998-2000) and BAKBASEL (years 2001–2011) for the most recent periods^13^.

Our primary source of wealth estimates are tax data. The main advantage of tax data over wealth surveys is that they have been compiled over a longer period and that they cover the entire population^14^. Moreover, tax series are easily comparable over time and contain a clear definition of wealth.

Federal wealth taxes have been levied intermittently between 1913 and 1957. The cantons have continued to tax wealth ever since. For 1913, 1919, 1969, 1981, 1991, 1997 and 2003–2011, detailed wealth tax data are available for the entire adult population with net worth above CHF 1000^15^. Based on those data, Dell et al. (2007) extrapolated population wealth estimates from the wealth of tax filers, assuming that non-filers’ share of wealth in years with incomplete data coverage is identical to their share in the closest year with complete coverage. We use their estimates for 1913–1997 and add the wealth-tax statistics for 2003–2011. We add wealth estimates for 1900 and 1910 based on the assumption that household wealth represented 80% of taxable capital^16^.

Wealth estimates based on wealth tax data have two main drawbacks, both biasing them downward. First, tax valuations of real estate correspond on average to some 70% of market values (see, e.g. Stutz et al. 2007). Second, pension fund assets are exempt from wealth taxes and therefore not covered by the tax data. However, an estimated 20–30% of pension assets are not annuitized but withdrawn upon retirement and therefore bequeathable^17^.

We address the issue of undervalued real estate by using data on net private wealth including real estate at market values published since 2004 by the Swiss National Bank^18^. Those data allow us to establish that, given gross real estate wealth is roughly equal to net wealth as measured through wealth taxes, a 30% undervaluation of real estate happens to imply a 30% underestimate of real wealth when based on tax data. As we have no reason to expect the degree of undervaluation of real estate to have increased over time, we consider back-projecting this 30% markup on tax-based wealth data all the way to 1911 to be a conservative adjustment^19^.

To quantify potentially bequeathable wealth inherent in pension funds, we use historical data on total pension fund assets reported by Leimgruber (2008) and corresponding data for 2011 by the Swiss National Bank. Based on unpublished data by the Swiss Federal Statistical Office, we can establish that since a liberalization in favour of lump-sum payouts in 2005, some 30% of pension assets have on average been paid out rather than annuitized^20^. Prior to the 2005 reform, lump-sum payouts were somewhat less common, in the order of 20% of total assets (Bütler and Teppa 2007). We therefore augment our estimated wealth series by 20% of aggregate pension assets in all years except for 2011, where we apply a share of 30%^21^.

### Age-wealth profiles

In order to compute *μ*_*t*_, the ratio between average wealth at death and average wealth of the living, we need age-wealth profiles either of decedents (as in Piketty 2011) or of the living. We can draw on age-wealth profiles of the living based on tax records for the canton of Zurich, covering the years 1934, 1945, 1969, 1975, 1987, 1995, 1999, 2003, 2005, 2010 and 2013. We approximate the 2011 value with the data for 2010. Since age-wealth profiles before 1934 are not available, we define *μ*_1911_ as the linear extrapolation of this ratio based on sample years 1934 to 1969, excluding the war years^22^.

Two corrections need to be made before calculating *μ*. First, for some years, the lowest age group covers ages 0 to 24 (1969-2003) or 0 to 29 (1934). To have homogeneous series covering the adult population aged 20 years or more, we apply linear extrapolation. For each year *t*_*m*_ where the 20–24 age bracket is missing, we take data from the closest year *t*_*c*_ with complete data. We assume that the 20–24 age group has the same proportion of taxpayers and wealth compared to the age bracket just above (e.g. 25–35 or 25–30) in both years *t*_*c*_ and *t*_*m*_, namely:

$$r_{W} = \frac{W_{t_{m},20}}{W_{t_{m},25}} = \frac{W_{t_{c},20}}{W_{t_{c},25}},\quad r_{T} = \frac{T_{t_{m},20}}{T_{t_{m},25}} = \frac{T_{t_{c},20}}{T_{t_{c},25}}. $$

Then, the data for the missing 20–25 bracket are estimated as follows:

$$W_{t_{m},20} = r_{W}* W_{t_{m},25}, \quad T_{t_{m},20} = r_{T} *T_{t_{m},25}. $$

Second, the Zurich age-wealth distributions show evident outlier values in years 1999–2010. The top wealth bracket of the 90+ age group for 2003–2010 (85–90 for 1999) has an unusually high average wealth compared to adjacent age groups. The explanation is the presence of Walter Haefner, a billionaire from Zurich who died aged 101 in 2012. In those years, his wealth was estimated at CHF3.3bn, and he was the world’s oldest billionaire^23^. We apply a linear correction similar to that for missing age brackets. In any year *t*_*h*_ where the presence of this exceptional individual likely skewed the data (1999–2010), we change the wealth owned by the top wealth bracket in the respective age group using data from the closest year *t*_*y*_ where this individual was not present. We assume that the ratio of the average wealth of the top wealth bracket *ω*_*top*_ to the average wealth of the adjacent lower wealth bracket *ω*_*second*_ of the relevant age group *a*_*H*_ is identical in years *t*_*h*_ and *t*_*y*_:

$$r_{w} = \frac{w_{t_{h},a_{H},\omega_{top}}}{w_{t_{h},a_{H},\omega_{second}}} = \frac{w_{t_{y},a_{H},\omega_{top}}}{w_{t_{y},a_{H},\omega_{second}}}, $$

$$w_{t_{h},a_{H},\omega_{top}} = r_{w} * w_{t_{h},a_{H},\omega_{second}}. $$

**Fig. 9:**
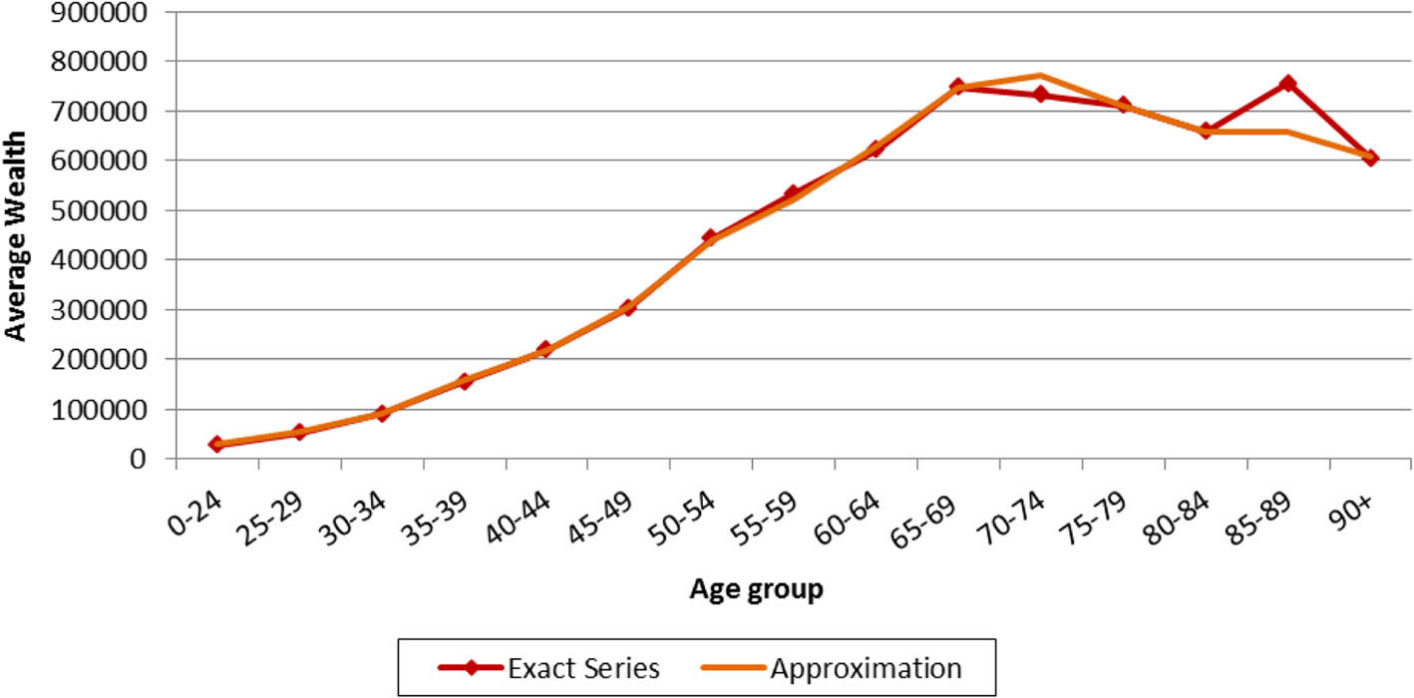
Average age-wealth profiles of the living in 1995 (canton of Zurich). Notes: In 1995 Swiss francs. See text for data sources

Not applying this correction would lead to estimated *μ*_*t*_ that are up to 21 percentage points higher in the period 1999–2011. Detailed data available for 1995 allow us to compare our approximated age-wealth profile with the age-wealth profile using the exact wealth per age group and wealth bracket. As shown in Fig. 9, the two age-wealth profiles turn out to be almost identical.

### Adult mortality

Another ingredient to our calculations are mortality rates *m*_*t*_, defined as the number of adult decedents over the adult population. We take those data from four sources. First, we use the Swiss adult population numbers from Dell et al. (2007) for the years 1900–2000. The number of decedents by age group for 1900–1991 is taken from Siegenthaler (1996). This series is updated with the BEVNAT database of the Swiss Federal Statistical Office and completed with the online database “Historical Statistics of Switzerland” hosted by the University of Zurich^24^.

For differential mortality rates of the rich and the poor, $m_{t}^{r}(a)$ and $m_{t}^{p}(a)$, we follow Piketty (2011) in assuming a constant differential over time, corresponding to the US-based estimates by Attanasio and Hoynes (2000). To the extent that American mortality differences across wealth classes are likely to exceed the corresponding differences in Europe, this choice implies that our estimates of *μ*_*t*_ will be conservative.

### Gifts *inter vivos*

For bequests not to be underestimated, *inter vivos* gifts need to be taken into account. No time-series information exists on this ratio for Switzerland, but we have a number of useful pointers to the size of this variable.

Based on cantonal tax data, Daepp (2003) estimated the gifts-to-bequests ratio *v*_*t*_ for a sample of cantons in the period 1995–2002. We show these estimates in Table 2. Daepp’s (2003) data point to a *v*_*t*_ of about one third in the late 1990s^25^.

To project this ratio back in time, we assume that it has tracked the evolution observed in Germany, using the estimates of Schinke (2012). We make this choice for two reasons. First, in the years for which we have data for both countries, German values of *v*_*t*_ are close to those for Switzerland. In 2002, for instance, the German gift-to-bequest ratio was estimated at 34%, very close to the numbers reported by Daepp (2003). Second, Germany seems to offer a better benchmark for backward projection than France, because its tax treatment of gifts and bequests has remained relatively stable, and life expectancy, the main demographic driver of *v*_*t*_, has increased at comparable rates in Switzerland and Germany (see Moreau 2013)^26^.

**Table 2:** The importance of inter vivos gifts *inter vivos* gifts in percent of the volume of bequests

Year	Zurich	Bern	Ticino	Vaud	Germany
	Source: Daepp (2003)				Source: Schinke (2012)
1911					8
1961					18
1973					30
1978					30
1995		30.6			
1996		41.8			
1997		42.2	42.4		
1998	49.1	39.3	31.1		
1998^a^	36.9				
1999		27.5	40.2		
2000		23.4			
2001		34.4		17.9	
2002		33.5		43.9	34
2007					58
2009					59
Average		33.5	36.5	30.4	

^a^Excluding wealth transfers larger than CHF 200 million

The German *v*_*t*_, however, increased sharply after 2002. We do not consider it plausible that the incidence of *inter vivos* gifts jumped in a comparable manner in Switzerland, which is why our baseline estimates will be based on a linear extrapolation of the prior evolution of estimated *v*_*t*_^27^. This implies a moderate increase in the ratio *v*_*t*_ over the most recent decade, consistent with the observed increase in life expectancy. Our imputed Swiss *v*_*t*_ for 2011 is 39%, instead of the 50% observed in Germany. We shall explore the robustness of our estimates to this assumption^28^.

### Capital shares and saving rates

In order to compute the inherited share of private wealth *ϕ* according to Eq. (3), we need data for *α*, the share of national income accruing to capital, and for *s*, the saving rate.

Capital shares from 1995 onwards are published by the Swiss Federal Statistical Office as a component of the national accounts. For 1910–1947, historical data compiled by researchers at the University of Zurich provide a credible and consistent series^29^. No data of comparable quality exist for the period 1948–1994. We therefore interpolate these years based on capital shares for Germany as reported by Alvaredo et al. (2017), as they track Swiss capital shares quite closely in the periods for which we have data in both countries^30^.

Saving rates from 1991 onwards are available from Eurostat. For 1948–1990, we can draw on comparable series from the Swiss Federal Statistical Office^31^.

## Footnotes

1 This is also what Dell et al. (2007) find for the evolution of wealth inequality over the last century: Switzerland did not follow the “u” of other European nations.

2 We largely retain Piketty’s (2011) notation. Piketty (2011) also computed an alternative measure labeled “fiscal inheritance flows”, derived from inheritance tax statistics. Since bequests have never been taxed at the federal level in Switzerland, and since we were unable to find sufficiently comprehensive data at the canton level, this alternative measure cannot (yet) be applied to the Swiss case.

3 $m_{t}=\frac {N_{dt}^{20+}}{N_{t}^{20+}}$, where $N_{dt}^{20+}$ is the number of adult deaths (20 or older), and $N_{t}^{20+}$ is the number of living individuals aged 20 or older.

4 In doing so, we follow Schinke (2012). Note that Piketty (2011) could observe wealth at death directly in French inheritance data.

5 See, e.g., Hurd and Smith (2001) or Kopczuk and Saez (2004).

6 We find $sh_{t}^{p}(a)$ to be 4.25% on average, with no evident time trend (see Moreau 2013). As wealth is distributed more equally within older age brackets, $sh_{t}^{p}(a)$ is higher for older groups. Ignoring age-differentiated $sh_{t}^{p}(a)$ would lead to an under-estimation of *μ* of up to 8.1 percentage points.

7 Think for example of a situation where total wealth *W*_*t*_(*a*)=1000 and the total population in the age bracket equals 100 (so that the average wealth is *w*_*t*_(*a*)=1000/100=10), with $W_{t}^{p}(a)=200$ and $W_{t}^{r}(a)=800$. Then $sh_{t}^{p}=200/1000=0.2$ and the average wealth of the poor $ w_{t}^{p}(a)=200/50=4$ which is indeed equal to $w_{t}^{p}(a)=2\cdot sh_{t}^{p}(a)\cdot w_{t}(a)=2\cdot 0.2\cdot 10$.

8 Compulsory private pension-fund assets (the “second pillar” of the Swiss old-age pension system) are exempt from wealth taxes and therefore not covered by tax data. Some 20-30% of these assets are on average withdrawn as lump-sum payouts rather than being annuitized. What remains of these assets at death becomes part of the bequeathable estate.

9 Figure 2 shows a spike in 1918, explained by the “Spanish flu” pandemic of that year. This outlier does not affect our estimates.

10 It might appear surprising that Switzerland exhibits a similar (though somewhat less pronounced) u-shaped evolution of the wealth-to income ratio to that observed for other industrialized nations, given that this pattern is most intuitively attributed to war-related wealth destruction. It should be noted, however, that even though Switzerland was largely spared from the ravages of war, Swiss-owned assets located abroad were affected as well. Moreover, the decline in wealth-to income ratios after World War II was driven by fast income growth - a phenomenon just as evident in Switzerland as in other advanced economies. Waldenström (2017) finds a similar u-shaped evolution for Sweden, which was also not directly involved in the two world wars.

11 The drivers and nature of the growing wealth-to-income ratio in Switzerland would merit further investigation. It would for instance be interesting to explore the importance of housing wealth for shaping this evolution, given that residential land is an exhaustible resource (Grossmann and Steger 2017). Consistent with the theory, available statistics from the Swiss National Bank show that the weight of net housing wealth in total net wealth has increased from from 25% in 2004 to 32% in 2014.

12 Research on Danish data suggests that bequests have increased wealth inequality overall but reduced the top-1% wealth share (Boserup et al. 2016). Similar results have been found for Sweden (Elinder et al. 2016).

13 NNI estimates for 1911 and 1969 are not available in our source data, but we can interpolate them from data on adjacent years.

14 Schinke (2012) compares the two types of data source in the German context. For a discussion of the advantages and disadvantages of tax data over surveys for the study of income distribution in Switzerland, see Foellmi and Martinez (2017).

15 The compilations for 1913 and 1919 are available in the *Annuaire Statistique de la Suisse* 1914 (p. 226) and 1920 (p. 395). The published statistics for the remaining years are accessible on the website of the Swiss Federal Tax Administration: https://www.estv.admin.ch/estv/de/home/allgemein/steuerstatistiken/fachinformationen/steuerstatistiken/ gesamtschweizerische-vermoegensstatistik-der-natuerlichen-person.html.

16 Data on taxable capital for these two years can be obtained from the *Annuaire Statistique de la Suisse*, 1920, p. 395. In 1913 and 1919, the ratio of household wealth to total taxable capital was respectively equal to 81% and 79%.

17 A third potential source of systematic downward bias is tax evasion, which could come to light after death. We have no way of quantifying the importance of this phenomenon, but the relatively high elasticities of taxable wealth with respect to wealth tax rates detected by Brülhart et al. (2017) suggest that it may be significant.

18 See https://data.snb.ch/en/topics/uvo/cube/frsekgevehup.

19 Low valuations of real estate by local authorities have been a matter of contention particularly by federal tax authorities for decades, and harmonization efforts have aimed at imposing minimum tax valuation thresholds in the cantons, currently officially at 60% of market value. Moreover, agricultural real estate, which tends to be undervalued even more strongly by tax authorities, has been more important as a share of wealth in the past, a factor further confirming the conservativeness of our assumption. The combined share of land and housing in total assets, however, seems to have remained relatively stable over time. Goldsmith (1981), for example, reports this share as 24.9% in 1938 and 23.5% in 1978.

20 We are grateful to Jerôme Cosandey and Willy Stuber for those data.

21 The available historical series for aggregate pension fund assets are not at an annual frequency. We therefore use linear interpolation to compute the required values for our sample years of interest.

22 Not considering years since 1969 avoids the sharp increase in this ratio over the last two sample decades, which is unlikely to be representative of the earlier trend.

23 Source: https://www.forbes.com/lists/2010/10/billionaires-2010/_Walter-Haefner/_FRD4.html, retrieved in January 2017.

24 See https://www.fsw.uzh.ch/histstat/.

25 We were able to cross-validate the reported ratios for Vaud with data obtained from the cantonal statistical office, and found Daepp’s (2003) numbers to be accurate. Stutz et al. (2007) estimate *v*_*t*_ as being at least 25% in the canton of Zurich in 1997.

26 In France, however, a pronounced increase in *v*_*t*_ over the last two decades is probably explained by changes in the tax code, making France a less suitable benchmark.

27 Schinke (2012, 29) explains the observed rise in gifts by stating that “public awareness for the issue of inheritance, taxes and ways to circumvent them has risen considerably during the time”. Given the long-standing nature of discussions about bequest taxation in Switzerland, an equivalent shift does not seem probable to us (see Brülhart and Parchet 2014). The exception is the year 2011, in which many gifts were made in anticipation of a retroactive clause in a pending initiative for the introduction of a federal estate tax.

28 Through another project we have access to the universe of individual-level tax records in the canton of Bern for the years 2001-2011. In those data, declared *inter vivos* gifts fluctuated around 0.75% of net wealth between 2001 and 2010, without a detectable trend (see also Jann and Fluder 2015). This corroborates our baseline assumption of *v*_*t*_=39% in 2011 (since *b*_*wt*_ is estimated as 2.7% in 2011, see Table 1). These data also confirm that 2011 was a non-representative outlier, with a gift-to-wealth ratio of 3.4% in Bern. Note, therefore, that our reported inheritance estimates for 2011 are based on extrapolated gift-to-wealth ratios and are unaffected by the one-off wave of *inter vivos* transfers in 2011.

29 See https://www.fsw.uzh.ch/histat/main.ph.

30 Up to 1994, national accounts statistics separately reported income shares of labor, capital and closely held corporations, the latter category comprising both capital and labor according to our definitions. The evolutions over time in the (partial) series for Switzerland and those for France and Germany match quite closely.

31 For 1980-1990, these data are compiled using the same accounting standards as the later Eurostat data (ESA1995). Prior to that, the accounting standard OECD64 is applied. For the purposes of our study, these series are comparable.
